# Molecular Mechanisms Mediating Retinal Reactive Gliosis Following Bone Marrow Mesenchymal Stem Cell Transplantation

**DOI:** 10.1002/stem.2095

**Published:** 2015-07-29

**Authors:** Alessia Tassoni, Alex Gutteridge, Amanda C. Barber, Andrew Osborne, Keith R. Martin

**Affiliations:** ^1^ Department of Clinical Neurosciences, John van Geest Centre for Brain RepairUniversity of CambridgeCambridgeUnited Kingdom; ^2^ Pfizer NeusentisGranta ParkCambridgeUnited Kingdom; ^3^ Cambridge NIHR Biomedical Research CentreCambridgeUnited Kingdom; ^4^ Eye DepartmentAddenbrooke's HospitalCambridgeUnited Kingdom; ^5^ Wellcome Trust—Medical Research Council Cambridge Stem Cell InstituteCambridgeUnited Kingdom

**Keywords:** Glia, Stem cell transplantation, Retina, Mesenchymal stem cells

## Abstract

A variety of diseases lead to degeneration of retinal ganglion cells (RGCs) and their axons within the optic nerve resulting in loss of visual function. Although current therapies may delay RGC loss, they do not restore visual function or completely halt disease progression. Regenerative medicine has recently focused on stem cell therapy for both neuroprotective and regenerative purposes. However, significant problems remain to be addressed, such as the long‐term impact of reactive gliosis occurring in the host retina in response to transplanted stem cells. The aim of this work was to investigate retinal glial responses to intravitreally transplanted bone marrow mesenchymal stem cells (BM‐MSCs) to help identify factors able to modulate graft‐induced reactive gliosis. We found in vivo that intravitreal BM‐MSC transplantation is associated with gliosis‐mediated retinal folding, upregulation of intermediate filaments, and recruitment of macrophages. These responses were accompanied by significant JAK/STAT3 and MAPK (ERK1/2 and JNK) cascade activation in retinal Muller glia. Lipocalin‐2 (*Lcn‐2*) was identified as a potential new indicator of graft‐induced reactive gliosis. Pharmacological inhibition of STAT3 in BM‐MSC cocultured retinal explants successfully reduced glial fibrillary acidic protein expression in retinal Muller glia and increased BM‐MSC retinal engraftment. Inhibition of stem cell‐induced reactive gliosis is critical for successful transplantation‐based strategies for neuroprotection, replacement, and regeneration of the optic nerve. Stem Cells *2015;33:3006–3016*

**Significance Statement**

Over the last decade there has been considerable interest in the potential use of stem cell therapy for protective, regenerative and cell replacement purposes in a variety of neurodegenerative conditions of the eye and brain. However a major barrier to overcome for clinical translational is the aggressive reactive gliosis which the host tissue undergoes in response to grafted stem cells in some systems. We dissected signalling pathways behind graft induced reactive gliosis and proposed a molecular mechanism orchestrating this response, in the context of MSC transplantation for optic nerve protection and repair. As reactive gliosis is a potential obstacle to stem cell‐based strategies throughout the CNS, our finding may have considerable relevance in the field of stem cell therapy for CNS protection and repair.

## Introduction

Retinal ganglion cells (RGCs) are the output cells of the retina and RGC axons transmit visual information from the eye to the brain via the optic nerve. The optic nerve can be affected by many pathological conditions, including traumatic, ischemic, inflammatory, and degenerative disorders 1. Different etiologies may result in progressive and irreversible loss of RGCs 1 resulting in partial or complete visual loss. Glaucoma, the leading cause of irreversible blindness worldwide, is a degenerative condition characterized by the selective loss of RGCs. We have previously shown the feasibility of protecting RGCs using bone marrow mesenchymal stem cells (BM‐MSCs) in a rat model of glaucoma 2, 3. However, one of the unaddressed obstacles is the gliotic response that astrocytes and Muller glia (non‐neuronal support cells within the retina) exhibit in response to grafted cells 4, 5, 6. In retinal cell neuroprotection and replacement therapy, the glial response to the donor cell graft is an important barrier to overcome in order to enhance synaptic plasticity and axonal remodeling of surviving neurons as well as integration of a variety of donor cell types into the host retinal circuitry 4, 5, 7, as shown in the outer 5 and inner retina 4, 8, 9.

Current solutions to attenuate the glial response are limited. Considering the widespread use of MSCs in current therapeutic research, a better understanding of glial responses to MSC transplantation and the signaling pathways that drive them is required, particularly with regard to the possible detrimental effects of glial cell reactivity. Furthermore, understanding of the host glial response following donor cell transplantation into the eye could be more broadly informative within the field of stem cell regenerative research for the development of more efficient and successful therapies. Indeed, reactive gliosis following transplantation is not limited to MSCs, but it occurs in response to many other donor cell types, including neuronal cells 9 induced pluripotent cells 10, 11, Muller stem cells 12, and photoreceptor precursors 5, transplanted either into the vitreous or in the subretinal space for regenerative and protective purposes. Moreover, similar hostile glial responses are not confined to the retina and the optic nerve but have also been observed in other part of the CNS, such as in the spinal cord following Schwann cell transplantation at the site of injury 13.

In this study, retinal glia responses to grafted BM‐MSCs and the associated molecular mechanisms were investigated. We report for the first time the characteristics of the strong inflammatory response that accompanies reactive gliosis following BM‐MSC transplantation and we identify LCN2 as an additional indicator of reactive gliosis in the recipient retina. Moreover, we suggest the MAPK and JAK/STAT3 cascades in Muller cells are central mechanisms orchestrating glial and inflammatory responses to donor BM‐MSCs.

## Materials and Methods

### Animals

C57BL/6 mice (6–8 weeks old) were purchased from Charles River, Ltd. (Kent, U.K., http://www.criver.com). Hes5‐GFP transgenic mice were provided by Prof. Verdon Taylor (University of Basel, Switzerland). Glial fibrillary acidic protein (GFAP)‐STAT3‐cKO mice were donated Prof. Michael Sofroniew (UCLA). All procedures were carried out in accordance with U.K. Home Office regulation for the care and use of laboratory animals and the U.K. Animals (Scientific Procedures) Act (1986).

### Cell Culture

The C57BL/6 mouse GFP^+ve^ BM‐MSC (Cyagen Biosciences, Inc., Sunnyvale, CA, http://www.cyagen.com/us/en/), MIO‐M1 (donated by Prof. Astrid Limb, UCL, London, U.K.), and Fibroblast NIH3T3 cell line (Cell Biolabs, Inc., San Diego, CA, www.cellbiolabs.com) were cultured in Dulbecco's modified Eagle's medium, 10% fetal bovine serum penicillin (100 U/ml), and streptomycin (100 µg/ml) at 37°C and 5% CO_2_ (all components from Life Technologies, Leicestershire, U.K., https://www.lifetechnologies.com). Mouse neural precursor cells (NPCs), donated by Dr. Stefano Pluchino (University of Cambridge, U.K.), were prepared and cultured as described 14.

### Intravitreal Stem Cell Transplantation

Mice (*n* = 5) were anesthetized and eyes were treated with topical administration of tetracaine hydrochloride 0.5% and tropicamide. Intravitreal injections (2 µl) of Zymosan or stem cells (10,000 cells per microliter) were performed with a 33 Gauge needle (Hamilton, Co., Reno, NV; http://www.hamiltoncompany.com) on a 5 µl syringe (Hamilton, Co., http://www.hamiltoncompany.com). Phosphate buffered saline (PBS) sham control injection was performed in the right eye. Animals were culled 7 days after transplantation.

### EdU Proliferation Assay

One day before sacrifice, animals (*n* = 3) were intraperitoneally injected with 50 mg/kg of EdU (5‐ethynyl‐2′‐deoxyuridine, Life Technologies, Leicestershire, U.K., https://www.lifetechnologies.com). In in vitro assays, EdU (10 µg/ml) was added to the medium 3 hours before cell fixation. EdU labeling was conducted using Click‐iT EdU Alexa Fluor 555 Imaging Kit according to the manufacturer's protocol.

### Proteome Profiler Array

The mouse cytokine array (R&D System, Abingdon, U.K., https://www.rndsystems.com) was used according to manufacturer's protocol. Retinal lysate membranes (*n* = 4) were developed using the ECL Prime Detection Reagent (Amersham, GE Healthcare, Little Chalfont, U.K., http://www3.gehealthcare.co.uk) and quantification by band densitometry using ImageJ.

### Muller Cell Isolation

Hes5‐GFP^+ve^ retinas were dissociated in a single‐cell suspension by 7‐minute incubation in 0.1% trypsin (Sigma‐Aldrich, Corp., Cambridge, U.K., https://www.sigmaaldrich.com/united-kingdom.html) at 37°C. DNase 0.01% (D5025‐15 ku, Sigma‐Aldrich, Corp., Cambridge, U.K., https://www.sigmaaldrich.com/united-kingdom.html) was added to the sample followed by centrifugation at 600*g* for 5 minutes. The pellet was resuspended in Mg^2+^/Ca^2+^‐free Hanks' balanced saline solution (Life Technologies), containing 1% bovin serum albumin (BSA) (Sigma‐Aldrich, Corp., Cambridge, U.K., https://www.sigmaaldrich.com/united-kingdom.html), 0.05% trypsin inhibitor (Sigma‐Aldrich, Corp., Cambridge, U.K., https://www.sigmaaldrich.com/united-kingdom.html), and 0.002% DNase. Samples were centrifuged and resuspended in 1% BSA at a density of 10^7^ cells per milliliter. GFP^+ve^ cells were sorted and collected in RNeasy Lysis Buffer (RLT) buffer for RNA extraction. Purity of fluorescence‐activated cell sorted (FACS) Muller cells was assessed by Power Syber Green RNA to Ct‐1 step kit (Applied Biosystems, Leicestershire, U.K., https://www.lifetechnologies.com) according to manufacturer's instructions. Primer sequences used in both these assays are listed in Supporting Information Table S1.

### Microarray Gene Expression Profiling

Retinal total RNA (naïve control *n* = 4; Zymosan, PBS, MSC injected *n* = 5) was extracted using the RNeasy micro kit (Qiagen, Manchester, U.K., https://www.qiagen.com) according to manufacturer's instructions. Genomic DNA was removed by DNase (Qiagen, Manchester, U.K., https://www.qiagen.com) treatment. RNA samples were amplified using the TotalPrep Amplification Kit (Applied Biosystems, Leicestershire, U.K., https://www.lifetechnologies.com). Biotin‐labeled cRNA (1.5 µg) was hybridized on the Illumina mouse WG‐6 v2.0 Expression Beadchip at 55°C for 18 hours. Hybridized BeadChips were then labeled with streptavidin‐Cy3 and scanned with the Illumina BeadStation 500 system. Microarray data were quantile normalized and log 2 transformed prior to analysis.

### Quantitative RT‐PCR

Total RNA (naïve control *n* = 4; Zymosan, PBS, MSC injected *n* = 5) was reverse‐transcribed into cDNA using a High Capacity RNA to cDNA kit (Life Technologies, Leicestershire, U.K., https://www.lifetechnologies.com) according to manufacturer's instructions. Real‐time PCR was performed using TaqMan Gene Expression PCR Master mix (Applied Biosystems Leicestershire, U.K., https://www.lifetechnologies.com). All selected assays are listed in Supporting Information Table S1.

### Western Blot

Retinas (naïve control *n* = 4; Zymosan, PBS, MSC injected *n* = 5) were homogenized mechanically in lysis buffer (Roche, West Sussex, U.K., https://www.roche.co.uk) containing a protease inhibitor EDTA‐free tablet (Roche, West Sussex, U.K., https://www.roche.co.uk) and phosphatase inhibitor cocktail (Thermo Scientific, Loughborough, U.K., http://www.thermoscientific.com/en/home.html). Protein samples were run on precast gels (Life Technologies, Leicestershire, U.K., https://www.lifetechnologies.com) and electrotransferred to polyvnylidene Difluoride (PVDF) membranes (Life Technologies, Leicestershire, U.K., https://www.lifetechnologies.com). PVDF membranes were blocked in PBST (5% dried skimmed milk, in 0.1 M PBS 0.2% Tween 20 (Sigma‐Aldrich, Corp., Cambridge, U.K., https://www.sigmaaldrich.com/united‐kingdom.html) at room temperature (RT) for 1 hour, incubated overnight with primary antibody at 4°C and for 1 hour at RT with secondary antibody horseradish peroxidase (HRP) conjugated. Signal was developed using the ECL Prime Detection Reagent (Amersham, GE Healthcare, Little Chalfont, U.K., http://www3.gehealthcare.co.uk).

### Retinal Explant Culture

Retinal tissue was obtained from healthy adult mice as previously described 15. The isolated retina (control *n* = 4; treated *n* = 5) was cut into four equal‐sized explants, placed on 12‐mm diameter filters (0.4 µm pore, Millipore, Watford, U.K., http://www.merck.co.uk/en/index.html) with the RGC side up and cultured in Neurobasal‐A medium, supplemented with B27 (2%), N2 (1%), l‐glutamine (0.8 mM), penicillin (100 U/ml), and streptomycin (100 µg/ml) (all components from Life Technologies, Inc., Leicestershire, U.K., https://www.lifetechnologies.com). For coculture experiments, 1 µl of stem cell suspension (750 cells per microliter) was added to the RGC surface on day ex vivo (DEV) 1 and maintained for 5 days at 35°C and 5% CO_2_.

### Immunohistochemistry

Retinal explant tissue was processed for immunohistochemistry as previously described 16. Tissue sections were blocked in PBS containing 4% normal goat serum (Life Technologies, Inc., Leicestershire, U.K., https://www.lifetechnologies.com) and 0.3% Triton X‐100 (Sigma‐Aldrich, Corp.) for 1 hour, followed by incubation overnight at 4°C with primary antibody (Supporting Information Table S2) and with the appropriate AlexaFluor‐conjugated secondary antibody for 2 hours at RT the following day. Nuclei were counterstained with 4,6‐diamidino‐2‐phenylindole (Life Technologies, Inc., Leicestershire, U.K., https://www.lifetechnologies.com). Images were processed using Leica software (LAS AF V2.6.0 Leica Microsystems GmbH) and Image J.

### Statistics

Data shown are the mean ± SEM. Statistical analysis was performed with GraphPad Prism using the unpaired Student's *t* test, two‐way, or one‐way ANOVA with Bonferroni or Tukey's post hoc test. For microarray analysis, data were quantile normalized and log 2 transformed prior to analysis. Probes detected in fewer than three samples (Illumina detection *p* < .05) were also discarded. All analysis was conducted in R/Bioconductor 17. Principal components analysis was performed using the pcaMethods package 18. Differential expression analysis was performed using limma 19 and gene set enrichment analysis by camera 20 using gene ontology (GO) annotations 21.

## Results

### BM‐MSC Transplantation Induces Extensive Reactive Gliosis and Inflammation in the Host Retina

**Figure 1:**
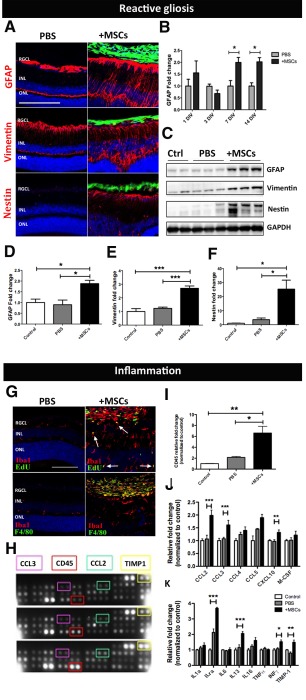
MSC transplantation induces reactive gliosis and inflammation in the recipient retina. **(A):** Immunostaining showing upregulation of the intermediate filaments (red) GFAP, Vimentin, and Nestin following transplantation. Scale bar = 200 µm. **(B):** Time course of GFAP protein expression in MSC recipient retinas relative to PBS sham injected retinas (*n* = 4, two‐way ANOVA). **(C):** Western blot and its quantification **(D–F)** confirming gliosis at 7 days post‐transplantation. **(G):** Immunolabeling for Edu and F4/80 (green) with the pan microglia marker Iba1 (red) showing microglia proliferation (white arrows) and macrophage recruitment in MSC recipient retina. Scale bar = 100 µm. **(H–K):** Proteome profiler array and its quantification (*n* = 4 per group). Black, gray, and white bars represent protein expression level in MSC recipient‐, PBS sham injected‐, and naïve control retina, respectively. Error bars represent SEM, *, *p* < .05, **, *p* < .01; ***, *p* < .001, one‐way ANOVA test with Tukey's correction. Abbreviations: GFAP, glial fibrillary acidic protein; INL, inner nuclear layer; MSC, mesenchymal stem cell; ONL, outer nuclear layer; PBS, phosphate buffered saline; RGCL, retinal ganglion cell layer.

Reactive gliosis has been reported to be a major barrier to effective stem cell transplantation therapy in the retina 4, 5. As shown by immunohistochemistry at 7 days post‐transplantation, GFAP was highly expressed in the Muller cell processes throughout the retinal layers of BM‐MSC recipient retinas, while its expression was limited to the astrocytes of the nerve fiber layer in sham control retinas (Fig. 1A). A similar glial response to transplanted cells was observed both in vivo and ex vivo using NPCs, MIO‐M1 cells, and fibroblasts (Supporting Information Fig. S1). Using Western blotting, we examined the temporal profile of the gliotic response following BM‐MSC transplantation at 1, 3, 7, and 14 days in vivo (DIV). GFAP expression significantly increased at 7DIV (Fig. 1B–1D) and remained elevated for at least 14 days, showing a 2.02‐fold (±0.17) increase in the host retina compared to PBS sham control (Fig. 1B). Alongside GFAP, both Nestin and Vimentin were significantly increased at 7DIV post‐BM‐MSC transplantation (Fig. 1A, 1C–1F).

Upon BM‐MSC transplantation, retinal tissue underwent structural disorganization characterized by an increase in the retinal thickness, retinal detachment, and folding of the outer nuclear layer (Supporting Information Fig. S2A, S2B). Retinal folding and detachment have previously been reported to be associated with excessive immune recruitment to the retina 22 and similar retinal changes can be observed after intravitreal injection of Zymosan, a proinflammatory mediator (Supporting Information Fig. S2C). We next investigated the inflammatory response following transplantation by measuring microglial reactivity and macrophage infiltration. EdU was administered intraperitoneally to mice 24 hours prior to sacrifice and retinas were double immunolabeled for Iba1 and EdU (Fig. 1G). In the presence of transplanted BM‐MSCs, Iba1^+ve^ cells were mainly distributed in proximity to the graft where they displayed a typical reactive hypertrophic phenotype. Moreover, the presence of cells positive for both Iba1 and EdU confirmed microglia proliferation in response to donor BM‐MSCs (Fig. 1G, white arrows). Conversely, in sham control eyes, microglial cells appeared to have a more scattered distribution and displayed the characteristic ramified morphology that is typical of microglia in a resting state (Fig. 1G). Microglial activation and macrophage infiltration were assessed using the marker F4/80 alongside Iba1 (Fig. 1G). Although no double positive labeling was observed within the retina of mice receiving PBS‐sham injections or BM‐MSC transplantation, strong reactivity for both F4/80 and Iba1 was detected within the BM‐MSC graft in the vitreous cavity, suggesting the recruitment of recipient‐derived infiltrating macrophages to the graft (Fig. 1G). To better understand macrophage chemotaxis induced by transplanted BM‐MSCs, the relative abundance of chemokines and cytokines released by the retina in response to grafted BM‐MSCs was investigated using a mouse cytokine array (Fig. 1H). The monocyte‐macrophage lineage marker CD45 showed a significant 6.6‐fold (±1.24) increase in the BM‐MSC transplanted group, compared to naïve control retina (Fig. 1I). Several chemokines and cytokines were also increased in response to grafted BM‐MSCs, with CCL2, CCL3, CXCL10, and INFγ showing a significant increase compared to naïve control retina (Fig. 1J, 1K). Results showed that following BM‐MSC transplantation, the host retina undergoes extensive reactive gliosis and inflammation, characterized by upregulation of intermediate filaments and blood stream‐derived monocyte recruitment.

### Gene Expression Profiling of BM‐MSC Recipient Retina

We next investigated possible signaling pathways orchestrating the gliotic and inflammatory response in the recipient retina following transplantation. Four experimental groups were examined: mice that received (a) intravitreally injected BM‐MSCs; (b) intravitreal PBS injections (sham control); (c) intravitreal proinflammatory Zymosan injections (positive control); and (d) naïve wild‐type retinas that were used as a negative control. Retinas were processed for gene expression profiling at 7 days postinjection. Principal component analysis was performed as an initial step in the analysis of the microarray to give an exploratory overview of the data. As observed in the plot (Supporting Information Fig. S3A), circles representing gene expression profile of each experimental group (red, turquoise, green, and purples circles indicating naive control, PBS sham control, BM‐MSC injected, and Zymosan injected group, respectively) cluster together, confirming similar gene expression profiling among biological replicates. Similar expression profiles of naïve wild‐type and the PBS sham injected controls confirmed that the surgical procedure did not have any major effect on the gene expression profile of these samples (red circles and turquoise circles, respectively). In order to identify and investigate the response of key signaling pathways following BM‐MSC transplantation, a GO‐based analysis of the microarray data was performed. GO terms representative of the JAK STAT and MAPK signaling cascade and their regulatory pathways were significantly enhanced in BM‐MSC receiving samples compared to PBS sham controls (Supporting Information Fig. S3B). Interestingly, in addition to the involvement of both the ERK1–2 and STAT3 signaling cascades, the P53‐mediated apoptotic signaling pathway was also significantly enriched in retinal samples receiving BM‐MSC transplants (Supporting Information Fig. S3B), suggesting a detrimental effect mediated by graft‐induced gliosis and inflammation.

**Figure 2:**
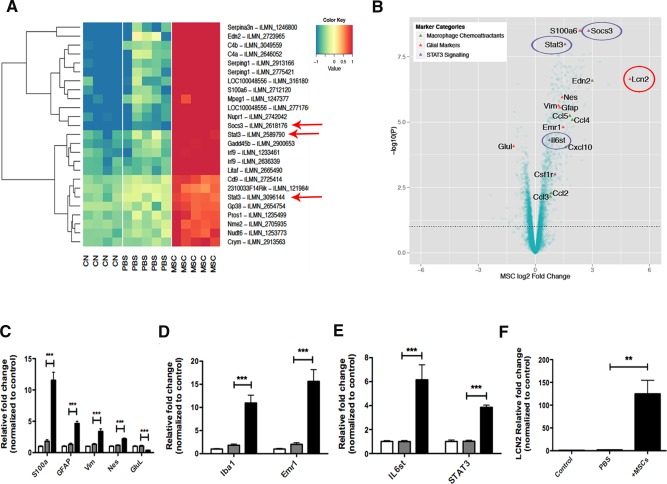
Microarray gene expression profiling of MSC recipient retina. **(A):** The top 25 probes showing the most significant changes in gene expression as ranked by ANOVA *p*‐value over the control (CN), sham (PBS), and MSC‐treated (MSC) groups. The expression levels of each probe across the three treatment groups are mean‐centered and are shown alongside the gene they map to. Red means high expression and blue means low expression compared to the average expression across the samples. Components of the STAT3 signaling are highlighted (red arrows). **(B):** Volcano plot showing key marker genes in retinal samples receiving MSC transplantation compared to PBS injected control groups. Key genes known to be markers of macrophage chemoattractant (green triangles), glial cells (red triangles), and STAT3 signaling (purple triangles) are highlighted. **(C–F):** Validation of the microarray data by qPCR. Error bars represent SEM, **, *p* < .01; ***, *p* < .001, one‐way ANOVA test with Tukey's correction. Abbreviations: CN, control; GFAP, glial fibrillary acidic protein; MSC, mesenchymal stem cell; PBS, phosphate buffered saline.

A heat map with the top 25 gene changes was generated in order to identify the most differentially expressed genes among the experimental groups. Interestingly, concomitant with the overexpression of glial and immune related genes, STAT3 and its endogenous suppressor SOCS3 were also demonstrated to be strongly upregulated in the BM‐MSC transplant group, suggesting the involvement of this pathway in the immune response of the retina to the graft (Fig. 2A, red arrows). A volcano plot comparing samples from PBS sham and BM‐MSC injected retina was performed in order to give an overview of genes whose expression altered the most (Fig. 2B). Alongside factors representative of gliotic response and immune cell recruitment, key members of the *STAT3* signaling pathway, including IL6st, STAT3, and SOCS3, appeared significantly induced in retinal samples receiving BM‐MSC transplantation (Fig. 2B, purple circles). Moreover, one of the genes that changed most in expression was the autocrine mediator of reactive astrocytosis *Lipocalin‐2* (*Lcn2*, Fig. 2B, red circle). Microarray data were validated by qPCR, where highly significant induction of glial markers, such as S100a, GFAP, Vimentin, and Nestin (Fig. 2C) and microglia/macrophage markers Iba1 and Emr1/F480 were observed (Fig. 2D). STAT3 and its upstream receptor IL6st (Fig. 2D) were also confirmed to be significantly upregulated in BM‐MSC recipient retina compared to naïve control, with a 6.1‐ (±1.25) and 3.85‐fold (±0.19) increase, respectively (Fig. 2E). Strikingly, a significant 125‐fold (±29) induction of *Lcn2* was demonstrated in BM‐MSC recipient retina (Fig. 2F, *p* = .0007). To assess STAT3 transcriptional activity in the Muller glia cell population, STAT3 induction was investigated in Muller glia isolated from BM‐MSC recipient retinae at 7 days postinjection. Isolation of a pure population of Muller cells was achieved by FACS of retinal cells from adult Hes5‐GFP^+ve^ mice. In these mice, green fluorescent protein (GFP) expression, driven by the Hes5 promoter, is lost during development and is restricted to mature Muller cells during adulthood (Supporting Information Fig. S4Ai–S4Aiii). The purity and identity of the sorted Hes5‐GFP^+ve^ cell population were confirmed by assessing the expression of marker specific for neurons (*Thy1* and *NeuN*), photoreceptors (*Recoverin Rcv*), astrocytes (*GFAP*), microglia (*Iba1*), and Muller glia (*Vimentin* and *GFP*) by PCR (Supporting Information Fig. S4B). Although a weak signal in *Recoverin* expression was observed, suggestive of the presence of photoreceptors contamination in the sorted cell population (Supporting Information Fig. S4B), the level of contamination was judged to be negligible. The gene expression level of *Rcv* and *GFP* in naïve retina and sorted Hes5‐GFP^+ve^ Muller cells was quantified and plotted in the bar graph in Supporting Information Figure S4C as percentage of expression relative to *Gapdh*. As reported, in naïve total retina the *GFP* and *Rcv* gene expression represented 0.47% ± 0.04% and 5.6% ± 1% of *Gapdh* expression, respectively. After sorting, in the Hes5‐GFP+ve cell population, the percentage of *Rcv* gene expression was reduced to 0.28% ± 0.1% of *Gapdh* expression, compared to the percentage of *GFP* expression, which increased to 20.23% ± 3.7% relative to *Gapdh* (Supporting Information Fig. S4C, white and black bars, respectively). Using this purified population of Hes5‐GFP^+ve^ Muller cells, gene expression was investigated by qPCR, confirming a 13.89‐ (±2.96), 38.93‐ (±2.13), and 2.21‐fold (±0.06) increase in *GFAP*, *LCN2*, and *STAT3* gene expression, respectively, in Muller cells following BM‐MSC transplantation (Supporting Information Fig. S4D–S4F).

### BM‐MSC Transplantation Results in LCN2 Production and Activation of STAT3 and ERK in Retinal Muller Glia

**Figure 3:**
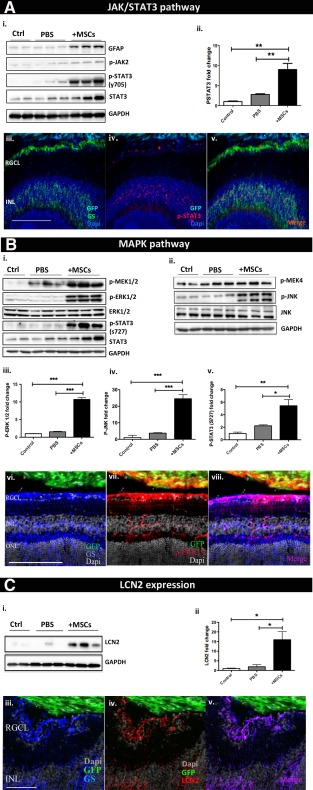
MSC transplantation results in LCN2 production and STAT3 and ERK activation in retinal Muller glia. Immunostaining and Western blot confirming activation of **(A)** JAK STAT cascade, **(B)** MAPK cascade, and **(C)** LCN2 in retinal Muller glia following MSC transplantation. Error bars represent SEM, *, *p* < .05; **, *p* < .01; ***, *p* < .001, one‐way ANOVA test with Tukey's correction. Abbreviations: GFAP, glial fibrillary acidic protein; GFP, green fluorescent protein; INL, inner nuclear layer; MSC, mesenchymal stem cell; ONL, outer nuclear layer; PBS, phosphate buffered saline; RGCL, retinal ganglion cell layer.

In the canonical JAK/STAT pathway, STAT3 activation involves phosphorylation of STAT3 on its tyrosine (Y705 p‐STAT3) residue 23. As assessed by Western blot at 7DIV (Fig. 3Ai), alongside GFAP upregulation, the total protein expression level of STAT3, as well as its phosphorylated state, was remarkably high in the BM‐MSC treatment group compared to the control and sham group, with phospho‐STAT3 showing a highly significant increase of 8.98‐fold (±1.5, *p* value = .0011) in retinas receiving GFP^+ve^ BM‐MSC transplants (Fig. 3Aii). Double immunolabeling for the Muller glia marker glutamine synthetase (GS) and p‐STAT3 confirmed activation of STAT3 in retinal Muller cells following transplantation (Fig. 3Aiii–3Av, green and red, respectively).

The microarray data also highlighted that the MAPK cascades, ERK1–2 and stress‐activated JNK, were among the most responsive pathways to BM‐MSC transplantation. MAPKs are serine/threonine kinases able to orchestrate cellular responses activating transcription factors by phosphorylation, for example, phosphorylating STAT3 on the Serine residue 727 24. Maximal transcriptional activity of STAT3 is achieved when STAT3 is phosphorylated on both tyrosine 705 and serine 727 25. To validate MAPK cascade activation, phosphorylation/activation of ERK1‐2 was examined and quantified by Western blot (Fig. 3Bi), showing a highly significant 10.71‐fold (±0.49, *p* < .001) increase following BM‐MSC transplantation (Fig. 3Biii). Alongside ERK‐1/2, activation of the stress‐activated JNK cascade was also confirmed (Fig. 3Bii), with JNK phosphorylation level undergoing a significant 24.51‐fold (±2.44, *p* value = .001) increase compared to naïve controls (Fig. 3Biv). This was accompanied by a significant 5.43‐fold (±0.93) increase in the phosphorylation on Ser 727 of STAT3 (Fig. 3Bv). Double immunolabeling for GS and phospho‐ERK1/2 confirmed activation of ERK1/2 in BM‐MSC recipient retinal Muller glia (Fig. 3Bvi–3Bviii, blue and red, respectively).

LCN2 was seen to be one of the most differentially expressed genes within the BM‐MSC recipient retina. An increase in the protein expression level of LCN2 at 7 DIV post‐transplantation was confirmed by Western blot (Fig. 3Ci), showing a 15.95‐fold (±4.1, *p* value = .009) increase in BM‐MSC transplanted retinas compared to naïve controls (Fig. 3Cii). Double immunolabeling of BM‐MSC transplanted retinal sections for the Muller glial marker GS and LCN2 confirmed LCN2 expression by Muller cells (Fig. 3Ciii–3Cv, blue and red, respectively). There is wealth of evidence that in response to upstream cytokines and growth factors, including IL6, CNTF, IL1β, and PDGF, both JAK and MAPK kinases induce STAT3 transcriptional activity by phosphorylating STAT3 on its tyrosine 705 and serine 727 residue, respectively 23, 26. Phosphorylated STAT3 dimers migrate into the nucleus and transcribe target genes, such as GFAP 27 and CxCl10 28. These results confirm that following BM‐MSC transplantation, the downstream effector of MAPK and JAK/STAT pathways STAT3 is significantly upregulated in retinal Muller glia. Such induction coincided with a remarkable increase in both LCN2 and GFAP gene expression level in isolated Muller cells, most likely induced by ERK1–2 29 and STAT3 27.

### Retinal Glia Undergo Reactive Gliosis Via a STAT3‐Dependent Mechanism Ex Vivo Which Limits Retinal Engraftment of Stem Cells

**Figure 4:**
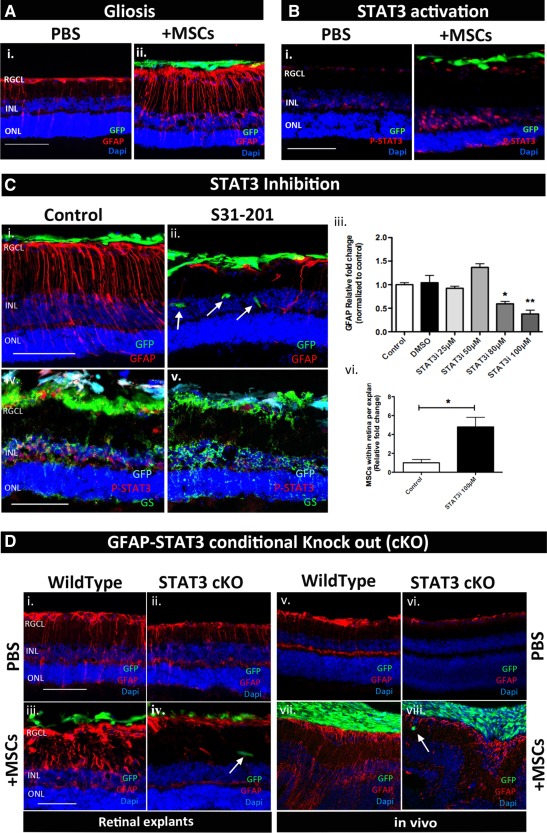
STAT3 as a central player in graft‐induced reactive gliosis ex vivo but not in vivo. **(A, B):** Immunostaining showing GFAP overexpression and STAT3 phosphorylation in retinal explants cocultured with MSC at 4 days ex vivo. **(C):** Immunostaining showing the effect of S31–201 in suppressing GFAP expression (i–iii), increasing MSC engraftment (ii, vi), and inhibiting STAT3 phosphorylation (iv, v). **(D):** Immunostaining showing that GFAP overexpression induced by donor MSCs is suppressed in GFAP‐STAT3 cKO mice ex vivo (i–iv) but not in vivo (vi–viii); *n* = 3 per group. Scale bar = 100 µm. Error bars represent SEM, *, *p* < .05; **, *p* < .01. Abbreviations: GFAP, glial fibrillary acidic protein; GFP, green fluorescent protein; INL, inner nuclear layer; MSC, mesenchymal stem cell; ONL, outer nuclear layer; PBS, phosphate buffered saline; RGCL, retinal ganglion cell layer.

The retinal explant organotypic culture system is an ex vivo model previously developed in our lab as a screening tool for neuroprotective therapies 15, 16. Here, we used the same model to test the efficiency of selected drugs in modulating glial reactivity following coculture with BM‐MSCs. For this purpose, GFP^+ve^ BM‐MSCs were cocultured on the inner surface of retinal explants for 4DEV. We first investigated whether the retinal organotypic culture system recapitulates the in vivo response to grafted BM‐MSCs. Similarly to what was observed in vivo, at 4DEV retinal explants undergo upregulation of GFAP and phosphorylation of STAT3 in the inner nuclear layer in presence of cocultured BM‐MSCs (Fig. 4A, 4B). In order to assess whether STAT3 plays a central role in graft‐mediated reactive gliosis in the explant system, we investigated the effect of pharmacological inhibition of STAT3 on GFAP overexpression following BM‐MSC coculture. Different doses of STAT3 inhibitor VI (S31–201) were tested; STAT3 inhibition at 80 and 100 µM successfully suppressed GFAP expression compared to vehicle treated samples (Fig. 4Ci–4Ciii). Interestingly, GFAP suppression mediated by the STAT3 inhibitor (100 µM) was accompanied by a significant increase of 4.8‐fold (±1.01) in the number of GFP^+ve^ BM‐MSCs infiltrating the host retinal layers, in comparison to control retinal explants where BM‐MSC migration within the tissue was very rare (Fig. 4Ciii, 4Cvi). Double immunofluorescence for phosphorylated STAT3 and the Muller glia marker GS confirmed inhibition of STAT3 in explants receiving the pharmacological agents (Fig. 4Civ, 4Cv). STAT3 inhibition did not affect neuronal survival and expression of the other intermediate filaments, vimentin and nestin (Supporting Information Fig. S5).

In order to confirm the central role of STAT3 in retinal Muller cell gliosis induced by grafted BM‐MSCs, retinal GFAP expression following coculture with BM‐MSCs was explored in retinal explants obtained from GFAP‐STAT3 conditional knockout mice (GFAP‐STAT3‐cKO), where conditional deletion of STAT3 from GFAP expressing cells was achieved using the Cre/loxP system 30. Unlike wild‐type retinal explants, STAT3cKO retinal explants cocultured with BM‐MSC did not show any increase in GFAP expression (Fig. 4Di–4Div). In addition, small numbers of BM‐MSCs were found within the host tissue (Fig. 4Div).

Finally, the role of STAT3 in GFAP overexpression following BM‐MSC transplantation was investigated in vivo using GFAP‐STAT3 cKO mice in comparison to wild‐type control animals. The overall expression of GFAP in Muller cells processes following PBS sham injection was reduced in cKO mice compared to WT retinas (Fig. 4D compare v with vi); however, cKO retinas did still show some upregulation of GFAP in response to transplanted BM‐MSCs (Fig. 4D compare vi with viii).

The observation that GFAP upregulation via STAT‐3‐dependent mechanisms is less marked in vivo than ex vivo suggests that in vivo other pathways beside STAT3 contribute to reactive gliosis. Therefore, we questioned whether LCN2 and ERK1/2, highly induced in vivo following BM‐MSC transplantation, were also upregulated in the retinal explants system. Surprisingly, neither activation of ERK1/2 nor increase in LCN2 protein expression level was observed in the presence of cocultured BM‐MSCs (Supporting Information Fig. S6). We therefore hypothesize that an important role might be played in vivo by recruited macrophages, whose role in glial activation and chronic inflammation is undetectable in an isolated system such as the retinal explant organotypic culture.

## Discussion

To date, the evidence of structural and functional benefits offered by transplanted MSCs to neurons in animal models of neurodegenerative conditions is very encouraging 31, 32, 33 and many clinical trials have been investigating the safety and efficacy of MSCs in a variety of neurodegenerative diseases, with promising preliminary results 34, 35, 36. As we recently published 2 in a rat model of glaucoma, transplantation of BM‐MSCs into the vitreous body resulted in a significant 30% increase in optic nerve axonal survival, supporting further investigation of the use of MSCs for neuroprotective purposes. Although neuroprotective strategies will not rescue dead RGCs, neuroprotection could conceivably slow down the progressive deterioration that many patients experience over the course of their disease. Moreover, rudimentary reconnection resulting from some level of plasticity achieved by stem cell‐based strategies might potentially be beneficial to patients with advanced visual loss.

However, despite the benefits conferred by MSCs to axonal survival, one of the major limitations that still needs to be overcome is the extensive reactive gliosis occurring in the recipient retina following MSC transplantation 4, 6. Reactive gliosis represents a barrier to successful stem cell‐based strategies in several neuropathological conditions 4, 5, 13, 37, 38. In an animal model of retinal degeneration both subretinal and intravitreal stem cell transplantation resulted in strong reactive gliosis and chondroitin sulfate proteoglycan deposition limiting stem cell migration into the host tissue 4, 5, 8, 12. This effect appeared to be independent of the type and origin of stem cells transplanted and, consistent with our study, was observed with both heterologous and autologous stem cell sources including Muller stem cells 12, photoreceptor precursors 5, neuronal progenitors 9, induced pluripotent stem cells 10, 11, and MSCs 4, 39. Here, we offer a detailed investigation of retina glial responses to intravitreal BM‐MSC transplantation in order to identify molecular mechanisms behind graft‐induced reactive gliosis in the retina.

We observed that following BM‐MSC transplantation recipient retina undergoes GFAP overexpression, extensive macrophage infiltration, and retinal folding and detachment. These responses were accompanied by activation of JAK/STAT3 and MAPK cascade and marked production of LCN2 by Muller glia. Using gene expression profiling, we identified LCN2, with a 125‐fold induction in gene expression in BM‐MSC recipient retina, as a potential new marker of retinal reactive gliosis. There is increasing evidence correlating astrocyte‐mediated LCN2 secretion to neuroinflammation 28, 40, reactive gliosis 41, and neuronal death 40. Although the mechanism through which LCN2 signals is not yet clear, its expression has been reported to be mediated by the ERK1/2 and INFγ/STAT1 signaling cascade 29. Moreover, in different models of neurodegeneration, LCN2 acts as chemokine inducer by promoting the upregulation of chemokines, such as CCL2 and CXCL10, through the JAK2/STAT3 pathway 28. There is evidence that under degenerative conditions reactive astrocyte gain neurotoxic properties and recent studies have identified LCN2 as an inducible neurotoxic mediator secreted by astrocytes to promote neuronal death 40. Based on this evidence, LCN2 secretion in the host retina following BM‐MSC transplantation suggests a potential detrimental glial response that needs to be modulated.

**Figure 5:**
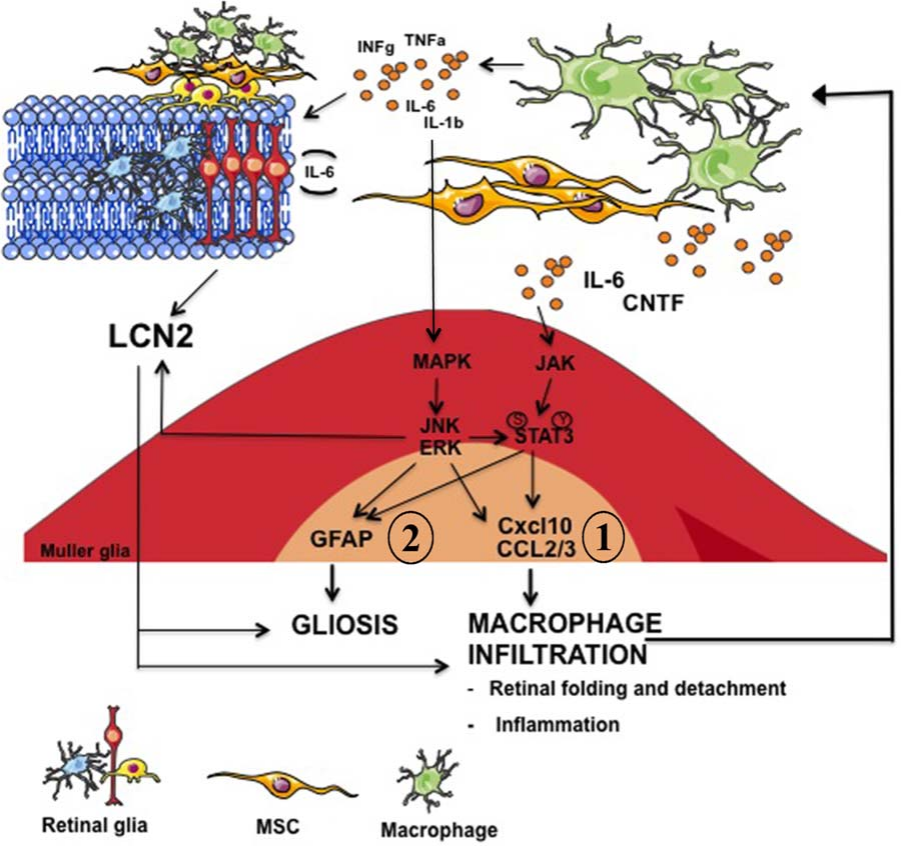
Schematic diagram depicting a possible molecular mechanism orchestrating retinal gliosis and neuro‐inflammation in response to donor MSCs. Abbreviations: GFAP, glial fibrillary acidic protein; MSC, mesenchymal stem cell.

Here, we propose a molecular mechanism orchestrating graft‐induced‐retinal reactive gliosis in the retina. As depicted in the diagram shown in Figure 5 (signaling 1) intravitreally transplanted BM‐MSCs secrete factors, including CNTF and IL6 3, 42, known to activate the JAK2/STAT3 cascade 3. Once activated, STAT3 mediates GFAP upregulation, as observed in our study and demonstrated previously 27, 30. According to recent studies 28, activated STAT3 is also involved in secretion of chemokines such as CCL2–3 and CXCL‐10, found to be highly upregulated in retinal tissue in response to BM‐MSC transplantation. Chemokines play a central role in macrophage recruitment. Activated macrophages invading the graft can secrete factors such as INFγ, IL‐1β, and IL6 which contribute to MAPK cascade activation 43 (signaling 2). This is supported by the evidence in our study that recipient retinas undergo MAPK activation in presence of BM‐MSCs in vivo but not in the macrophage‐free ex vivo system. The MAPK cascade may lead to further chemokine secretion 44, GFAP expression through the AP1/CREB complex 45, 46, and LCN2 expression 29, which we found to be significantly upregulated in the host retina in vivo but not ex vivo. Thus, we propose that LCN2 plays a central role in maintaining reactive gliosis 41 and neuroinflammation 28.

This proposed mechanism could explain why in STAT3 cKO animals in vivo we still observe some GFAP upregulation, in contrast to what we observed in retinal explants. One of the main differences between ex vivo and in vivo models is the absence of monocyte/macrophage infiltration ex vivo. In this case, it is likely that GFAP upregulation would be mediated solely by the JAK/STAT3 cascade potentially initiated by BM‐MSC‐secreted CNTF and IL6 (Fig. 5, signaling 2). The lack of a compensatory molecular pathway mediated by macrophage‐induced MAPK activation and LCN2 secretion (Fig. 5, signaling 2) could explain why ex vivo but not in vivo, knocking down STAT3 prevents retinal explants from upregulating GFAP. This is supported by our findings, where ex vivo the presence of donor BM‐MSCs cocultured on top of the retinal tissue is accompanied neither by ERK activation or by LCN2 (Supporting Information Fig. S6). Future studies will investigate the role of the proposed mechanism in BM‐MSC‐induced reactive gliosis in an animal model of experimental glaucoma in order to ensure that dampening down Muller glial response to grafted cells does not prevent BM‐MSC‐mediated neuroprotection.

## Conclusions

Our proposed mechanism provides a novel platform for further investigation into methods to attenuate recipient glial activation following cell transplantation. So far, neuroprotection appears a promising approach to enhance RGC survival and preservation of vision and may be more readily translatable to the clinic compared to RGC replacement or regeneration. However, the long‐term effects of reactive gliosis and its modulation need to be better understood before MSC transplantation could be considered as a potential neuroprotective therapy. Given that the retina and the optic nerve are part of the CNS, we suggest our findings may give some insights into mechanisms behind glial scar‐like barrier formation in response to transplanted cells potentially relevant to other parts of the CNS.

## Author Contributions

A.T.: conception and design, collection and/or assembly of data, data analysis and interpretation, and manuscript writing; A.G.: data analysis and interpretation; A.C.B. and A.O.: conception and design and manuscript writing; K.R.M.: conception and design, financial support, administrative support, manuscript writing, and final approval of manuscript.

## Disclosure of Potential Conflicts of Interest

The authors indicate no potential conflicts of interest.

## Supporting information

Supplementary Information Figure

Supplementary Information Figure 1

Supplementary Information Figure 2

Supplementary Information Figure 3

Supplementary Information Figure 4

Supplementary Information Figure 5

Supplementary Information Figure 6

Supplementary Information Table S1, S2
