# Supplementary material for: Molecular Mechanisms Mediating Retinal Reactive Gliosis Following Bone Marrow Mesenchymal Stem Cell Transplantation
Source: Stem Cells. 2015 Jul 29;33(10):3006–16. doi: 10.1002/stem.2095 (PMC4832383; doi:10.1002/stem.2095)
Supplement: Supplementary file 1 — Supplementary Information Figure [file STEM-33-3006-s001.doc]

Fig. S1: Reactive gliosis and preservation of retina structure was investigated following transplantation with a variety of stem cell sources by immunolabelling of retinal cross sections for GFAP (*red*). Nuclei were counterstained with DAPI (*blue*). Compared to controls, MSC transplantation resulted in GFAP over-expression, retinal detachment, folding of the outer layer and deformation of the inner retina (A *i-ii*). Similar responses were observed after intraocular injection of NPCs and fibroblasts (A *iii-iv*). Same result was observed *ex-vivo* (B), where co-culture with NPCs and MIO-M1 induced an increase in retinal GFAP similar to the one observed following co-culture with MSCs (B *i-iv*). Scale bar=100 μm.

Fig. S2: Reactive gliosis and preservation of retina structure after stem cell transplantation was investigated by immunolabelling of retinal cross sections for GFAP (*red*), Nuclei were counterstained with DAPI (*blue*). Compared to controls, MSC transplantation resulted in GFAP over-expression, retinal detachment, folding of the outer layer and deformation of the

inner retina (A-B). Similar responses were observed after intraocular injection of the proinflammatory inducer zymosan (C), confirming that the retinal structural alterations observed after stem cell transplantation was determined by ongoing reactive gliosis and inflammation (c). Scale bar=100 μm.

Fig. S3: Microarray gene expression profiling of MSC recipient retina. (A) Principal component analysis of treatment groups. Each point represents a microarray profile generated from one independent biological sample plotted according to the first two principal components identified from the data and coloured according to treatment group. (B) All GO signalling pathways (child terms of GO:0035556 (intracellular signal transduction)) that show a significant coordinated change in gene expression (camera FDR < 0.1) in response to MSC transplantation are presented in this heat map. The negative log10 camera P-value is shown in each case for the comparison of the MSC transplantation group with retinal samples receiving PBS sham (MSC, column 1); and comparison of naïve wildtype control samples with samples receiving PBS sham (CN, column 2).

Fig. S4: (A) Hes5-GFP^+ve^ retina in postnatal and adult mice (A *i-iii*) (B) Hes5-GFP+ve Muller cell were FACS sorted for GFP and purity of the sorted cell population was assessed by PCR. Isolated Hes5-GFP+ve cell populations showed negative for neuron (*Thy1, NeuN*), microglia *(Iba1*) or astrocytes ( GFAP) contamination. However, a faint band for Recoverin (*Rcv*) suggests the presence of a small proportion of photoreceptors. Positive bands for Vimentin in parallel to GFP confirmed the identity of sorted retinal Hes5-GFP+ve Muller cell population. (C) qPCR was performed in order to assess the level of photoreceptor contamination in isolated

Hes5-GFP+ve Muller cell population. Gene expression was normalized to *Gapdh* and plotted as the percentage of expression relative to *Gapdh* prior to and after sorting. These data ensure that

photoreceptor contamination in the Hes5-GFP+ve sorted cells is negligible after FACS sorting for GFP and the isolated cells represent a pure population of retinal Muller cells. (D-F) STAT3, GFP and LCN2 gene expression induction in recipient isolated Muller glia. *Black, grey and white* *bars* representing MSC recipient-, PBS sham injected- and naïve control retina, respectively. ** p<0,01, *** p<0,001.

Fig. S5: The effect of STAT3 inhibition on expression of glial markers Vimentin and Nestin was investigated in wildtype retinal explants 5 days after MSC co-culture. 100 μM STAT3 inhibition had no effect on Vimentin (A,B, *red*) or Nestin expression (C-D, *red*). Neuronal survival and retinal explant viability upon treatment with the STAT3 inhibitor was investigated by immunohistochemistry for NeuN (*blue*) and the apoptotic marker, activated caspase 3 (*red*) (E,F). No difference in the total number of NeuN+ve cells was detected between retinal explant receiving pharmacological treatment compared to those receiving PBS (G). In addition, no difference in the percentage of apoptotic neurons within the RGCL was detected (H, n=6 per group, black and white bars, respectively). Nuclear staining with DAPI showed that retinal architecture upon drug treatment was preserved and that STAT3 inhibition caused no changes in retinal thickness compared to PBS controls (I,J black and white bar, respectively). Grey =DAPI. Scale bar=100 μm.

Fig. S6: LCN2 expression and ERK activation in retinal explants after co-culture with MSCs.

No increase in protein expression level of LCN2 and phospho-ERK were observed across the experimental groups. GAPDH signals confirmed that protein samples had been loaded evenly.
