# Supplementary figures and images for: Molecular Mechanisms Mediating Retinal Reactive Gliosis Following Bone Marrow Mesenchymal Stem Cell Transplantation
Source: Stem Cells. 2015 Jul 29;33(10):3006–16. doi: 10.1002/stem.2095 (PMC4832383; doi:10.1002/stem.2095)

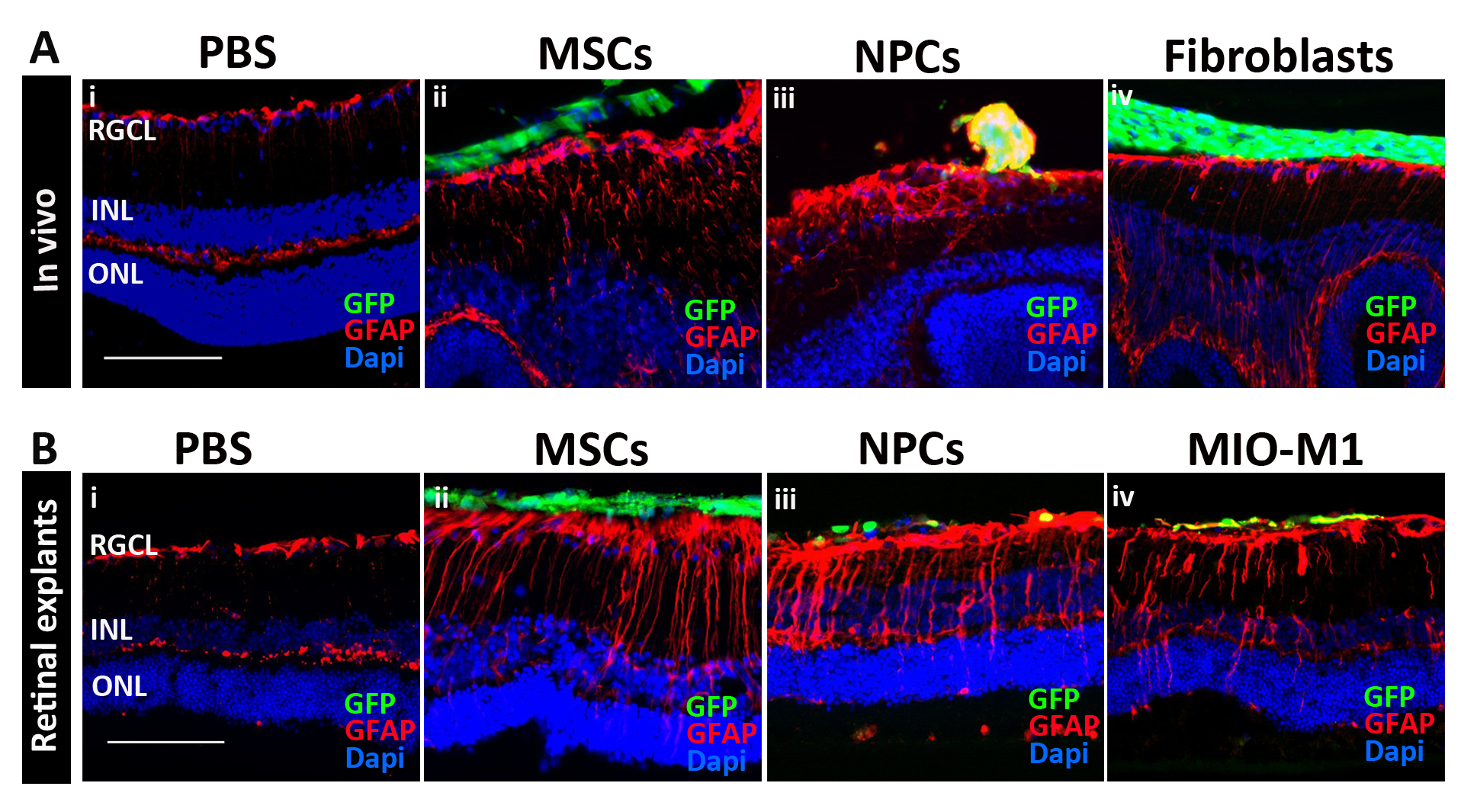

Supplement: Supplementary file 2 — Supplementary Information Figure 1 [file STEM-33-3006-s002.tif]

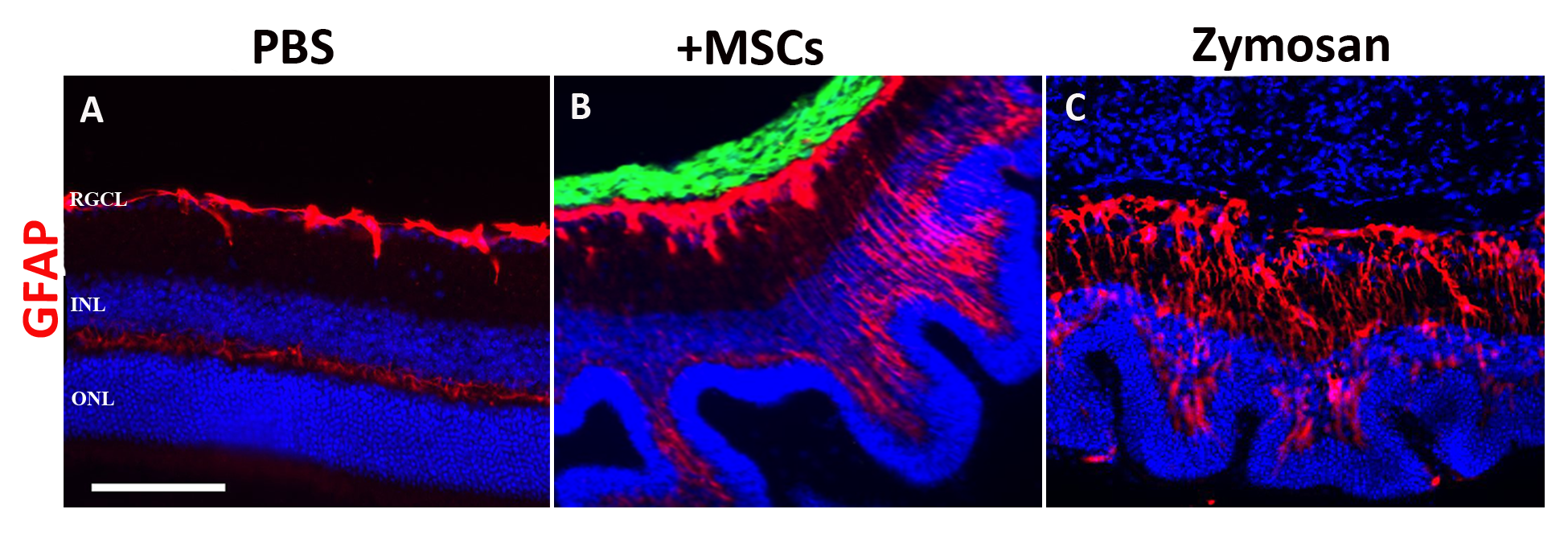

Supplement: Supplementary file 3 — Supplementary Information Figure 2 [file STEM-33-3006-s003.tif]

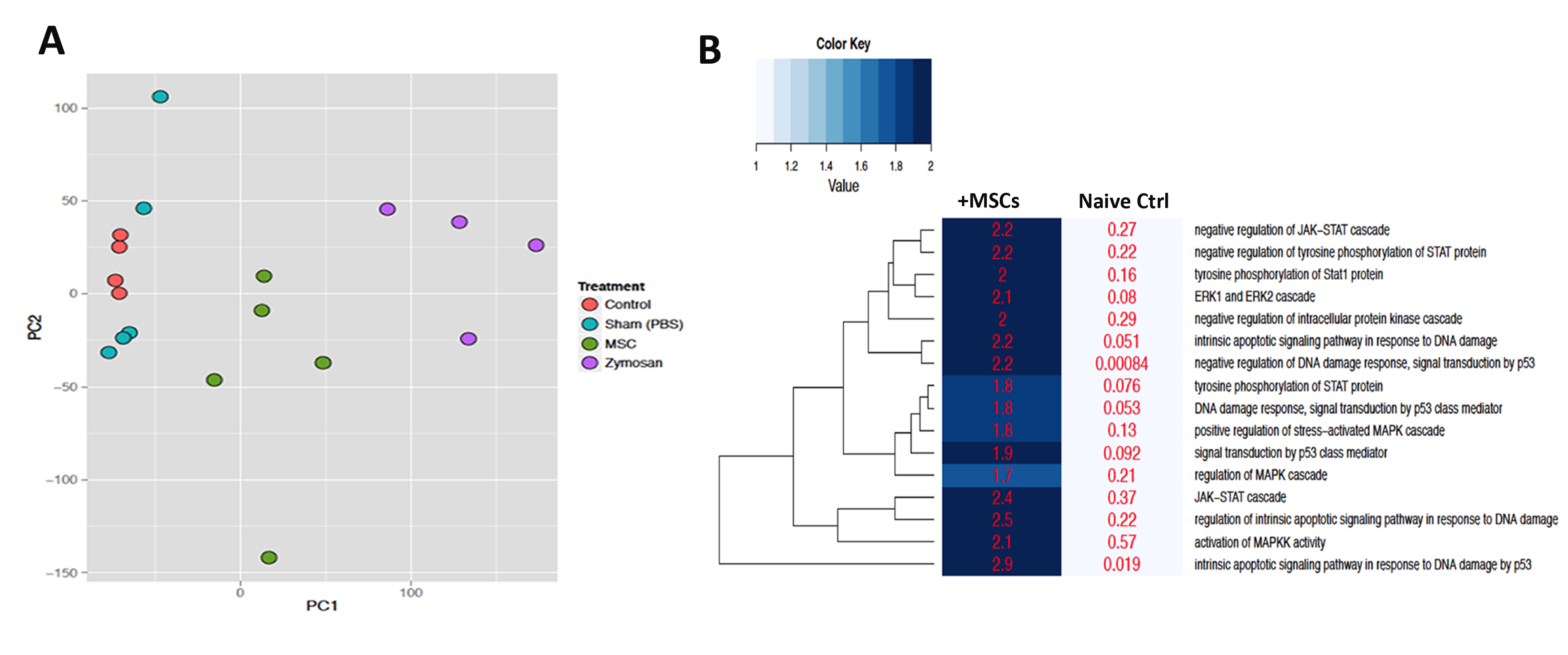

Supplement: Supplementary file 4 — Supplementary Information Figure 3 [file STEM-33-3006-s004.tif]

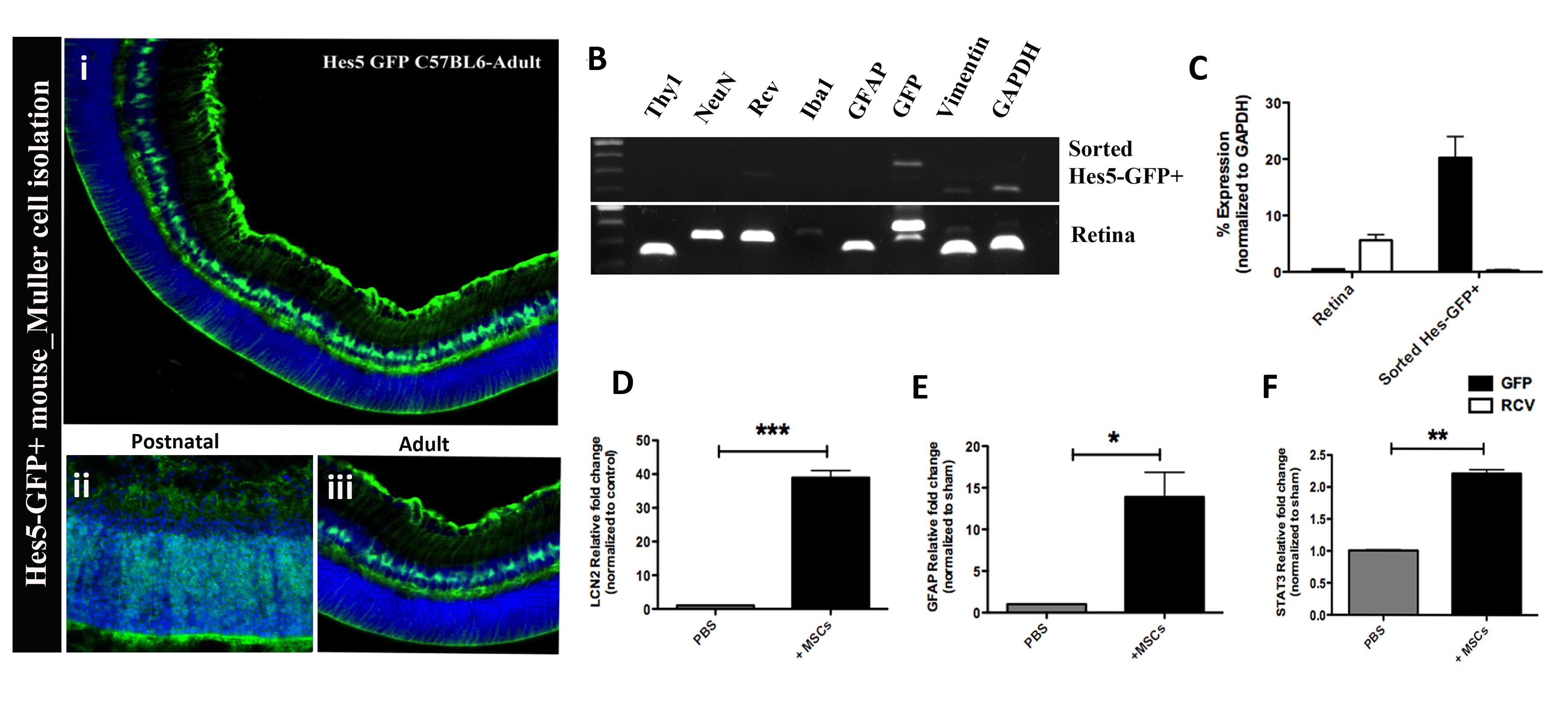

Supplement: Supplementary file 5 — Supplementary Information Figure 4 [file STEM-33-3006-s005.tif]

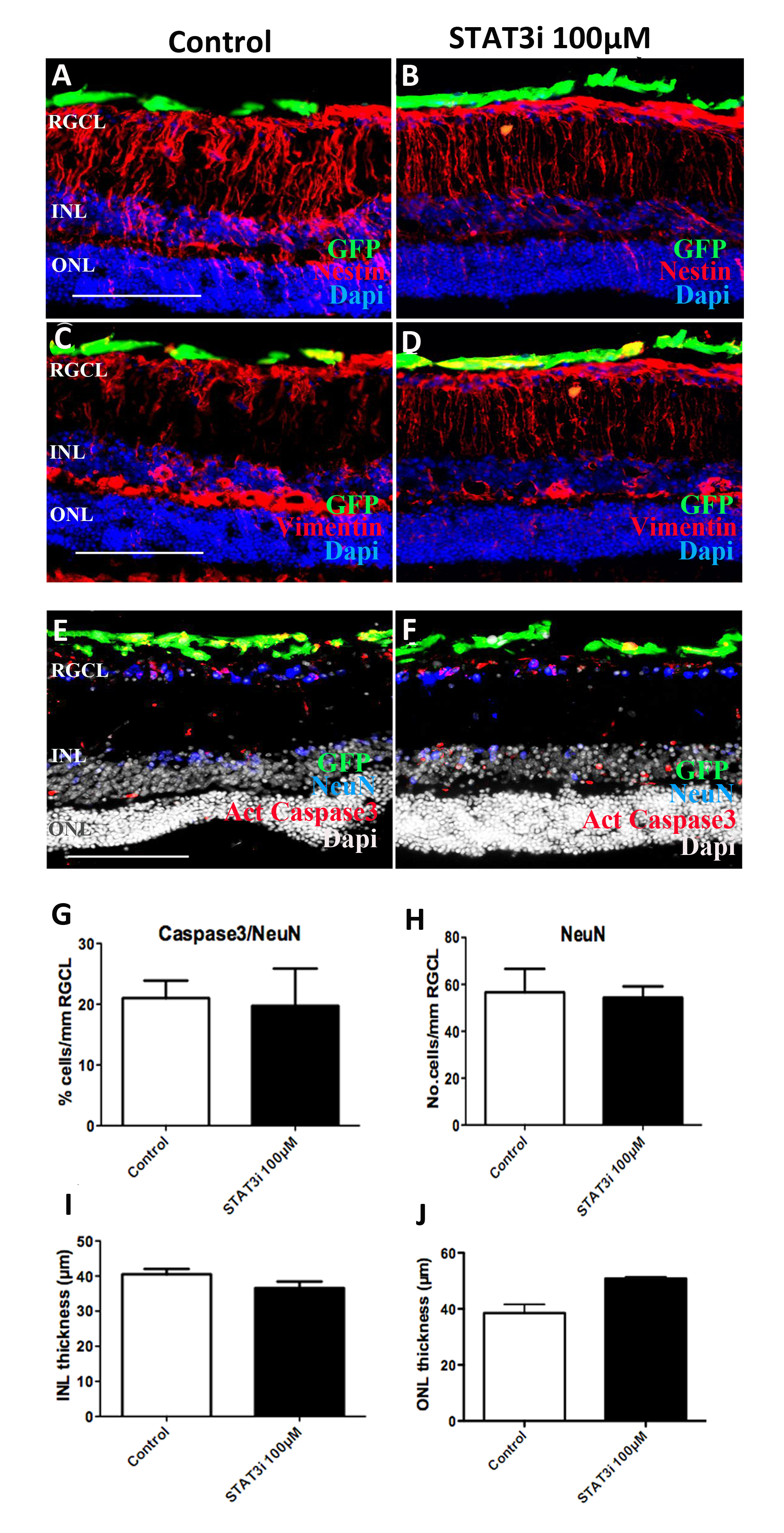

Supplement: Supplementary file 6 — Supplementary Information Figure 5 [file STEM-33-3006-s006.tif]

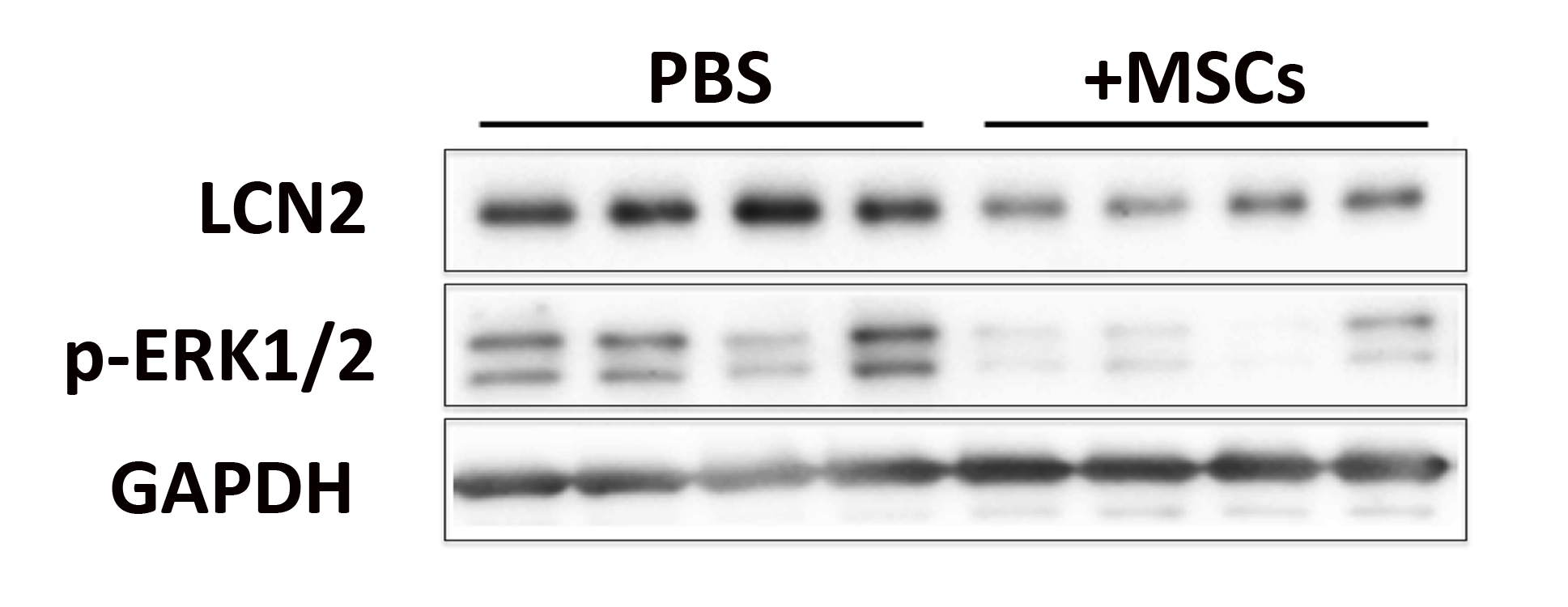

Supplement: Supplementary file 7 — Supplementary Information Figure 6 [file STEM-33-3006-s007.tif]
