# Supplementary material for: Molecular Mechanisms Mediating Retinal Reactive Gliosis Following Bone Marrow Mesenchymal Stem Cell Transplantation
Source: Stem Cells. 2015 Jul 29;33(10):3006–16. doi: 10.1002/stem.2095 (PMC4832383; doi:10.1002/stem.2095)
Supplement: Supplementary file 8 — Supplementary Information Table S1, S2 [file STEM-33-3006-s008.docx]

T**ABLE S1: Details of Primers used for One-step RT-PCR and Taqman Real Time PCR**

| **Gene** | **Forward** | **Reverse** |
| --- | --- | --- |
| **NeuN** | CCAGTCTTCCTTTCCCACCC | TTCTCACATAGGGGAGCGGA |
| **Thy1** | CCAATGAGGATGAGGGCTTA | GCAGGCTCGTGTTTTAGAGG |
| **Rcv** | AAGCGGGCTGAGAAGATCTG | CAGGGAGAGGTTTGGTTCCC |
| **Iba1** | GGACAGACTGCCAGCCTAAG | GTTTCTCCAGCATTCGCTTC |
| **GFAP** | CACGAACGAGTCCCTAGAGC | ATGGTGATGCGGTTTTCTTC |
| **GFP** | AGCTCGCCGACCACTACCAG | TCCCGACGCATCTTCTCCAC |
| **Vimentin** | ATGCTTCTCTGGCACGTCTT | AGCCACGCTTTCATACTGCT |
| **Gapdh** | AACTTTGGCATTGTGGAAGG | ACACATTGGGGGTAGGAACA |
| **S100a6** | Mm00771682_g1 (Life Technologies) | |
| **GFAP** | Mm01253033_m1 (Life Technologies) | |
| **Vimentin** | Mm01333430_m1 (Life Technologies) | |
| **Nestin** | Mm00450205_m1 (Life Technologies) | |
| **GluL** | Mm00725701_s1 (Life Technologies) | |
| **Iba1** | Mm00479862_g1 (Life Technologies) | |
| **Emr1** | Mm00802529_m1 (Life Technologies) | |
| **IL6st** | Mm00439665_m1 (Life Technologies) | |
| **STAT3** | Mm01219775_m1 (Life Technologies) | |
| **Lcn2** | Mm01324470_m1 (Life Technologies) | |

**TABLE S2: Primary antibodies**

| Specificity | Source | Isotype | Company | Concentration |
| --- | --- | --- | --- | --- |
| GFAP | Rabbit | Polyclonal | DAKO | 1:500 (IF) |
| GFAP | Mouse | Monoclonal | Sigma-Aldrich | 1:1000 (WB) |
| Vimentin | Chicken | Polyclonal | Chemicon | 1:500 (IF) |
| Vimentin | Rabbit | Polyclonal | Cell Signalling | 1:1000 (WB) |
| Nestin | Mouse | Monoclonal | Sigma Aldrich | 1:500(IF)  1:1000 (WB) |
| GS | Mouse | Monoclonal | BD Biosciences | 1:300 (IF) |
| Iba1 | Rabbit | Polyclonal | Wako | 1:400 (IF) |
| F4/80 | Rat | Monoclonal | AbD Serotec | 1:100 (IF) |
| GFP | Chicken | Polyclonal | Millipore | 1:5000 (IF) |
| P-ERK1/2 (Thr202/Tyr204) | Rabbit | Polyclonal | Cell Signalling | 1:500 (IF)  1:1000 (WB) |
| ERK1/2 | Rabbit | Polyclonal | Cell Signalling | 1:1000 (WB) |
| P-MEK1/2 | Rabbit | Polyclonal | Cell Signalling | 1:1000 (WB) |
| P-MEK4 | Rabbit | Polyclonal | Cell Signalling | 1:1000 (WB) |
| P-JNK | Rabbit | Polyclonal | Cell Signalling | 1:1000 (WB) |
| JNK | Rabbit | Polyclonal | Cell Signalling | 1:1000 (WB) |
| P-STAT3  (Tyr705) | Rabbit | Monoclonal | Cell Signalling | 1: 100 (IF)  1:1000 (WB) |
| p-STAT3 (Ser727) | Rabbit | Monoclonal | Cell Signalling | 1:1000 (WB) |
| STAT3 | Rabbit | Monoclonal | Cell Signalling | 1:1000 (WB) |
| LCN2 | Goat | Polyclonal | R&D System | 1:50 (IF)  1:1000 (WB) |
| NeuN | Mouse | Monoclonal | Millipore | 1:500 (IF) |
| Cleaved Caspase3 | Rabbit | polyclonal | Synaptic System | 1:500 (IF) |
| GAPDH | Mouse | Monoclonal | Sigma-Aldrich | 1:10000 (WB) |
